# Expression of *Vitis amurensis NAC26* in Arabidopsis enhances drought tolerance by modulating jasmonic acid synthesis

**DOI:** 10.1093/jxb/erw122

**Published:** 2016-05-07

**Authors:** Linchuan Fang, Lingye Su, Xiaoming Sun, Xinbo Li, Mengxiang Sun, Sospeter Karanja Karungo, Shuang Fang, Jinfang Chu, Shaohua Li, Haiping Xin

**Affiliations:** ^1^Beijing Key Laboratory of Grape Sciences and Enology, Laboratory of Plant Resources, Institute of Botany, Chinese Academy of Sciences, Beijing 100093, China; ^2^Key Laboratory of Plant Germplasm Enhancement and Specialty Agriculture, Wuhan Botanical Garden/Sino-Africa Joint Research Center, Chinese Academy of Sciences, Wuhan 430074, China; ^3^Department of Cell and Development Biology, College of Life Science, State Key Laboratory of Plant Hybrid Rice, Wuhan University, Wuhan 430072, China; ^4^National Center for Plant Gene Research (Beijing), Institute of Genetics and Developmental Biology, Chinese Academy of Sciences, Beijing 100101, China

**Keywords:** Drought stress, JA biosynthesis, ROS, transcriptional regulation, *VaNAC26*, *Vitis amurensis*.

## Abstract

The growth and fruit quality of grapevines are widely affected by abnormal climatic conditions such as water deficits, but many of the precise mechanisms by which grapevines respond to drought stress are still largely unknown. Here, we report that *VaNAC26*, a member of the NAC transcription factor family, was upregulated dramatically during cold, drought and salinity treatments in *Vitis amurensis*, a cold and drought-hardy wild *Vitis* species. Heterologous overexpression of *VaNAC26* enhanced drought and salt tolerance in transgenic Arabidopsis. Higher activities of antioxidant enzymes and lower concentrations of H_2_O_2_ and O_2_^−^ were found in *VaNAC26*-OE lines than in wild type plants under drought stress. These results indicated that scavenging by reactive oxygen species (ROS) was enhanced by *VaNAC26* in transgenic lines. Microarray-based transcriptome analysis revealed that genes related to jasmonic acid (JA) synthesis and signaling were upregulated in *VaNAC26*-OE lines under both normal and drought conditions. VaNAC26 showed a specific binding ability on the NAC recognition sequence (NACRS) motif, which broadly exists in the promoter regions of upregulated genes in transgenic lines. Endogenous JA content significantly increased in the *VaNAC26*-OE lines 2 and 3. Our data suggest that *VaNAC26* responds to abiotic stresses and may enhance drought tolerance by transcriptional regulation of JA synthesis in Arabidopsis.

## Highlight

*VaNAC26*, a member of the *NAC* gene family from the wild species *Vitis amurensis*, plays an important role in drought tolerance by positively modulating jasmonic acid synthesis and enhancing the antioxidant system.

## Introduction

Grapevine (*Vitis* species) is a deciduous woody perennial cultivated throughout the world across arid and semi-arid areas. The yield and berry quality of grapevines depends on vine adaptability to water deficits in water-limited environments. Regulated water deficit stress is widely used as part of viticulture management to balance vegetative and reproductive growth for improving berry quality (Lovisolo *et al.*, 2010). Moreover, most wine grapes are grown in regions with a Mediterranean climate where little rainfall is received during the growing season. Understanding the regulatory mechanisms underlying water deficit stress could inform the use of agronomic practices to improve grape productivity and quality (Romero *et al.*, 2012).

Mechanisms relating to how plants respond to drought stress have been widely studied in model plants such as Arabidopsis and rice (Kuromori *et al.*, 2014; Nakashima *et al.*, 2014). Drought stress activates the expression of a series of stress-related genes, especially transcription factors (TF). Depending on the involvement of abscisic acid (ABA), the drought-related transcriptional regulatory network can be divided into two main groups, an ABA-dependent and an ABA-independent pathway. TFs that belong to the AREB/ABF, MYB, MYC and NAC groups represent the major ABA-dependent pathway, while DREB, NAC and HD-ZIP TFs represent the major ABA-independent drought signal transduction pathway (Shinozaki and Yamaguchi-Shinozaki, 2007; Kuromori *et al.*, 2014). These TFs regulate the expression of downstream genes, which establish drought-stress tolerance in plants (Kuromori *et al.*, 2014).

NAC [*N*o apical meristem (NAM), *A*rabidopsis transcription activation factor 1/2 (ATAF 1/2), *C*UP-SHAPED COTYLEDON 2 (CUC 2)] proteins belong to a plant-speciﬁc transcription factor superfamily (Olsen *et al.*, 2005). *NAC* family genes contain a conserved sequence known as the DNA-binding NAC-domain in the N-terminal region and a variable transcriptional regulatory C-terminal region (Olsen *et al.*, 2005). NAC proteins have been reported to be associated with diverse biological processes, including development (Hendelman *et al.*, 2013), leaf senescence (Liang *et al.*, 2014) and secondary wall synthesis (Zhong *et al.*, 2006). In addition, a large number of studies have demonstrated that NAC proteins function as important regulators in various stress-related signaling pathways (Puranik *et al.*, 2012). The involvement of *NAC* TFs in regulation of a drought response was first reported in Arabidopsis. The expression of *ANAC019*, *ANAC055* and *ANAC072* was induced by drought and their overexpression significantly increased drought tolerance in transgenic Arabidopsis (Tran *et al.*, 2004). Following this study, a number of drought-related *NAC* genes have been identified in various species, such as *OsNAP* in rice (Chen *et al.*, 2014), *TaNAC69* in wheat (Xue *et al.*, 2011), and *ZmSNAC1* in maize (Lu *et al.*, 2012). This improved drought tolerance was found to partly result from regulation of the antioxidant system machinery. *OsNAP* was reported to decrease H_2_O_2_ content, and many other *NAC* genes (e.g. *NTL4*, *OsNAC5*, *TaNAC29*) have been found to regulate the antioxidant system (by increasing antioxidant enzymes or reducing levels of reactive oxygen species, ROS) under drought stress in different species (Song *et al.*, 2011; Lee *et al.*, 2012; Huang *et al.*, 2015). Moreover, several drought-related *NAC* genes have also been reported to be involved in phytohormone-mediated signal pathways, such as those for ABA, jasmonic acid (JA), salicylic acid (SA) and ethylene (Puranik *et al.*, 2012). For example, *ANAC019* and *ANAC055* were induced by ABA and JA, while *SiNAC* was identified as a positive regulator of JA and SA, but not ABA, pathway responses (Tran *et al.*, 2004; Puranik *et al.*, 2012).

In grapevines, the physiological and biochemical responses to drought stress have been primarily investigated with respect to such aspects as photosynthesis protection, hormonal variation and metabolite accumulation (Stoll *et al.*, 2000; Hochberg *et al.*, 2013; Meggio *et al.*, 2014). Transcriptomic, proteomic and metabolomic profiles have also been investigated in grapevines under water deficit conditions (Cramer *et al.*, 2007; Vincent *et al.*, 2007). Several TFs, such as *CBF* (*VvCBF1/2/3*), *ERF* (*VpERF1/2/3*) and *WRKY* (*VvWRKY11*) have been shown to respond to drought stress but the regulatory mechanisms remain elusive (Xiao *et al.*, 2006; Liu *et al.*, 2011; Zhu *et al.*, 2013). The involvement of *NAC* TFs in regulation of the stress response has also been detected in grapevines, and two stress-related *NAC* genes have been cloned, including *VpNAC1* from *V. pseudoreticulata* and *VvNAC1* from *V. vinifera*. *VpNAC1* was regarded as a positive regulator in the fungal-stress response (Zhu *et al.*, 2012), while *VvNAC1* was reported to be involved in both organ development and biotic and abiotic stress responses (Le Hénanff *et al.*, 2013).

In our previous study, a total of 74 *NAC* genes were identified from the 12× *V. vinifera* ‘Pinot Noir’ genome (Wang *et al.*, 2013). Among them, *VvNAC26* showed the greatest changes in expression under water deficit, cold temperature, and high salinity stresses in public microarray data. We cloned the coding sequence (CDS) of *VaNAC26* from *V. amurensis* (a cold- and drought-hardy *Vitis* species; Xin *et al.*, 2013; Su *et al.*, 2015). qRT-PCR results showed significantly increased transcription levels of *VaNAC26* under low temperature, drought, and high salinity treatments. Transgenic plants with heterologous overexpression of *VaNAC26* in Arabidopsis were generated, and the possible roles of *VaNAC26* during abiotic stresses were evaluated. At the same time, physiological and transcriptomic changes in transgenic plants under drought stress were carefully analysed. The data reported here suggest that *VaNAC26* responds to abiotic stresses and may enhance drought tolerance by transcriptional regulation of JA synthesis in Arabidopsis.

## Materials and methods

### Plant material and growth conditions

Tissue culture plantlets of *V. amurensis* [collected from Changbai Mountain (43^o^ N) in Jilin province, Northeastern China] were grown on 1/2 B5 medium (Gamborg *et al.*, 1968) with 30g L^−1^ sucrose, 0.2mg L^−1^ IAA, 0.7% agar, and 0.058% 2-(N-morpholino) ethanesulfonic acidhydrate (MES) in a growth chamber (16-h light / 8-h dark) at a constant temperature of 26 ^o^C. Plantlets with five well-developed leaves were subjected to abiotic stresses.

*Arabidopsis thaliana* ecotype Columbia (Col-0) was used in both wild type (WT) and transgenic experiments. Plants were grown in soil in a greenhouse with 16-h white fluorescent light (120 μmol m^–2^ s^–1^) / 8-h dark photoperiod at 22 ^o^C.

### Coding region and phylogenetic analysis of *VaNAC26*

The coding region of *VaNAC26* in *V. amurensis* was cloned based on annotated transcripts of GSVIVT01019952001 in the 12× *V. vinifera* ‘Pinot Noir’ genome (quasi-homozygous line PN40024, http://www.phytozome.net). The deduced amino acid sequences of VaNAC26 were used for searching homologous proteins by the BLASTp program in the GenBank database (http://www.ncbi.nlm.nih.gov/). Multi-alignment of VaNAC26 with five NAC proteins in Arabidopsis was performed by using DNAMAN software (http://www.lynnon.com/). A phylogenetic tree was constructed by the neighbor-joining (NJ) method using the MEGA5 program with Poisson-corrected distances, with 1000 bootstrap replicates.

### Subcellular localization of VaNAC26

To construct a *VaNAC26*::eGFP vector, the ORF sequence of the *VaNAC26* gene without terminator code TGA was cloned into the pCAMBIA1302 vector at BGLII/SpeI to obtain a fusion vector. After sequencing confirmation, the construct and empty vectors were transiently transformed into *Nicotiana benthamiana* leaves according to a previous protocol (Sheludko *et al.*, 2007). Infected cells of the lower epidermis of transformed leaves were analysed at 72h after inoculation. Confocal imaging was performed using a FLUOVIEW FV1000 laser scanning confocal microscope (Olympus, Japan). Post-acquisition processing of images was done using the Zeiss FV1000 Viewer 3.0 software (Olympus, Japan). GFPuv was excited at 488nm and emitted through a 505–550nm bandpass filter. DAPI was excited at 405nm and emitted at 500–600nm.

### Transactivation assay of VaNAC26

The different coding region sections of *VaNAC26* were sub-cloned into the GAL4 DNA-binding domain of the pGBKT7 vector including the predicted DB domain (DNA binding) and AD domain using the in-fusion HD Cloning kit (Clontech Laboratories, Inc., USA) to produce seven plasmids of pGBKT7-*VaNAC26*a-g (Clontech Laboratories, Inc.,USA). Y2HGold yeast cells harboring pGBKT7-*VaNAC26*a-g were streaked on SD/-Trp and SD/-His/-Ade media in plates to observe yeast growth at 30 ^o^C for 3–4 d. A stained assay was performed by adding 20mg L^−1^ X-α-gal into SD/-His/-Ade medium.

### Abiotic stresses and chemical treatment of grapevine plantlets

For the low-temperature treatment, grapevine plantlets were transferred to another chamber with the same light/dark periods as above with a constant temperature of 4 ^o^C. For drought, salt, and ABA treatments, the plantlets were transferred to liquid medium with an additional 6% polyethylene glycol (PEG) 6000 (–0.2MPa), 100mM NaCl (−0.45MPa), or 100 μM ABA, respectively. The shoot apex with one well-developed leaf was harvested from three independent replicates of each treatment at 2, 4, 8, 24, and 48h after initiating treatments. Untreated leaves were collected before each treatment was initiated and are indicated as 0h samples. All samples were frozen in liquid nitrogen and stored at −80 ^o^C for subsequent total RNA isolation and real-time RT-PCR analyses.

### Overexpression of *VaNAC26* in Arabidopsis

The full-length cDNA of *VaNAC26* was sub-cloned into the pCAMBIA 1301s vector promoted by the CaMV35S promoter. The constructs were transferred into *Agrobacterium tumefaciens* GV3101, and then used to transform Col-0 Arabidopsis using the ﬂoral dip method described by Clough and Bent (1998). Seeds of the T0 and T1 generation were screened on MS agar medium (Murashige and Skoog, 1962) containing 50mg L^−1^ hygromycin (HPT). Positive transgenic plants were selected according to their segregation ratio (resistant:sensitive = 3:1) on HPT-containing medium, and were confirmed by genomic PCR. The T3 generation transgenic lines that displayed 100% resistance to HPT were considered homozygous, and thus were harvested individually for further analyses.

### Drought and salt tolerance assays of transgenic Arabidopsis

For drought and salt tolerance assays, three T4 generation transgenic lines (OE-1, 2 and 3) and wild type Arabidopsis were used. For the drought treatment, seedlings of *VaNAC26*-OE lines and WT were grown in soil at 22 ^o^C for 21 d. After irrigation, the phenotypes of each plant were observed during the following 10 d without watering. Then, plants were re-watered and recovered for 3 d. The drought treatment experiments were repeated six times for transgenic lines and wild type Arabidopsis with ten plants in each repeat, and soil water content was measured using a soil moisture recorder (L99-TWS-1, Fotel, China) at designated time intervals throughout the drought period. The final survival rates of both transgenic and WT plant were calculated. Fully expanded leaves were collected at specified days after drought treatment for both transgenic and WT plants for subsequent microarray, real-time RT-PCR, and physiological index determinations.

For salt tolerance analyses, three transgenic lines and wild type Arabidopsis were germinated on Petri dishes (90mm) on MS solid medium (at least 100 seeds for each line). After 7 d, the germinated seedlings were transferred to solid MS medium with 120mM NaCl for the following 15 d. The survival rates of each line were calculated based on three replicates.

### RNA extraction and reverse transcription

Total RNA was extracted from 100mg samples comprised of the shoot apex with one young fully expanded leaf using Column Plant RNAout 2.0 (Tiandz Inc., Beijing, China). To remove contaminating DNA, 10 µg total RNA was treated with RQ1 DNase (Promega, Madison, Wisconsin, USA). First-strand cDNA was synthesized from DNase-treated RNA using Superscript III reverse transcriptase (Invitrogen, Carlsbad, CA, USA) and diluted 20-fold for real-time PCR analysis.

### Quantitative Real-time PCR

In order to detect the expression pattern of *VaNAC26* in *V. amurensis*, prepared cDNAs from cold, drought, and salt treatments were amplified. The expression levels of *VvActin-7* (GeneBank accession no. XM_002282480) and *VvGADPH* (GeneBank accession no. XM_002263109) were used as reference genes simultaneously. All the primer sequences are listed in Supplementary Table S1 at *JXB* online.

The expression levels of *VaNAC26* in a transgenic Arabidopsis line were detected and cDNAs were generated from 21 d-old leaves of OE-1, 2, 3, and WT. To confirm the expression of putative *VaNAC26* downstream genes in Arabidopsis, cDNAs were generated from leaves of OE lines and WT before drought (0 d) and 5 d after applying the drought treatment. The primer pairs were designed for 11 genes, namely *COR15A* (At2g42540), *PDF1.2* (At5g44420), *PR5* (At1g18250), *LTP3* (At5g59320), *LTP4* (At5g59310), *BMY1* (At4g15210), *SWEET4* (At3g28007), *NATA1* (At2g39030), *MYB47* (At1g18710), *COR414-TM1* (At1g29395), and *14A* (At3g28290). *Actin2* (GeneBank accession no. AK318637) and *UBQ10* (GeneBank accession no. NM_001084884) were used as reference genes. All the primer sequences are listed in Supplementary Table S1.

The qRT-PCR reaction contained 1.0 µL of cDNA, 5.0 µL of 2× SYBR Green Mix (Roche, Basel, Switzerland), 0.4 µL of 10mM primer mix, and 3.6 µL of deionized water. Three biological and three technical replicates were performed for each sample. All qRT-PCR assays were performed on a StepOne Plus real-time PCR Instrument (Applied Biosystems, CA, USA), and the data was analysed using Qbase software.

### Analysis of electrolyte leakage, chlorophyll content, chlorophyll *a* fluorescence, and photosynthetic gas exchange parameters

Electrolyte leakage (EL) and chlorophyll content were measured using leaves from control conditions and from drought treatments at 8 d. EL was determined according to Su *et al.* (2015). Chlorophyll content was measured by dimethyl sulfoxide (DMSO) extraction following a modified method of Wellburn (1994). Chlorophyll *a* fluorescence and photosynthetic gas exchange parameters were determined using leaves from control conditions and from drought treatments at 4 and 7 d. Chlorophyll fluorescence measurements were tested with a portable fluorometer PAM-2500 (Walz, Germany) according to Su *et al.* (2015), and photosynthetic gas exchange parameters were determined using a Li6400 portable photosynthesis system (Li-COR, USA) with a 2×3cm leaf cuvette with a red–blue LED light source as described by De Angeli *et al.* (2013).

### Antioxidant enzymes and lipid peroxidation assay

To extract antioxidant enzymes, leaf samples of about 0.2g were ground and homogenized in 4mL ice-cold sodium phosphate buffer (50mM, pH 7.8) containing 1% polyvinylpyrrolidone. The homogenate was centrifuged at 12 000 *g* for 15min at 4 ^o^C. The supernatants were used as the crude extract for measurement of superoxide dismutase (SOD) (EC 1.15.1.1) and peroxidase (POD) (EC1.11.1.7) activities and the malondialdehyde (MDA) content assay.

The SOD activity was assayed by its ability to inhibit the photochemical reduction of nitro blue tetrazolium (NBT) (Giannopolitis and Ries, 1977). The POD activity was measured based on guaiacol oxidation (Chance and Maehly, 1955). The lipid peroxidation level was assessed by measuring the thiobarbituric acid (TBA)-reactive substances with a lipid peroxidation MDA assay kit (S0131, Beyotime, China).

### *In situ* histochemical localization of H_2_O_2_ and O_2_^−^

*In situ* accumulation of H_2_O_2_ and O_2_^−^ were detected by histochemical staining with diaminobenzidine (DAB) and nitro blue tetrazolium (NBT), respectively. For localization of H_2_O_2_, leaves were sampled and immediately vacuum-infiltrated in DAB solution with a DAB color development kit (P0202, Beyotime, China). For O_2_^−^ detection, another set of leaves were vacuum-infiltrated in a 1mg mL^−1^ NBT solution in 10mM phosphate buffer (pH 7.8). For both DAB and NBT staining, the infiltrated leaves were incubated at room temperature for 8h, and then transferred to 70% ethanol to deplete chlorophyll and visualize the brown and blue spots for H_2_O_2_ and O_2_^−^, respectively.

### Microarray analysis

Leaves from WT and three transgenic lines were collected before and after 5 d of drought stress. An equal amount of leaves from three independent transgenic lines that were harvested on the same day was pooled as OE lines for RNA isolation. Four samples were collected at 10.00h, which included WT 0 d, OE 0 d, WT 5 d and OE 5 d, and each sample was represented by two replicates.

Total RNA was extracted using TRIzol reagent (Invitrogen, USA). Chip hybridization and microarray analysis were performed using Affymetrix Microarray Services (CapitalBio Co., Beijing, China) (Shi *et al.*, 2014). For array hybridization, 200ng of total RNA was used for first-strand and second-strand cDNA synthesis. The cRNA was labelled with a biotinylated ribonucleotide analogue and was fragmented with fragmentation buffer using the MessageAmp™ Premier RNA Amplification Kit (Ambion, #1792, USA). After purification, 12.5 μg of labelled and fragmented cRNA probes were hybridized to the Arabidopsis arrays with the Hybridization, Wash and Stain Kit (Affymetrix, #900720, USA).

The arrays were scanned using a GeneChipR Scanner 3000 (Affymetrix, #3000, USA) (Shi *et al.*, 2014). The identification of differentially expressed genes was based on the fold change >2 or <0.5 with *P*-values <0.05. Pathway enrichment analysis was performed using the Classification SuperViewer Tool (http://bar.utoronto.ca/ntools/cgi-bin/ntools_classification_superviewer.cgi) (Provart and Zhu, 2003). Microarray data have been submitted to the Gene Expression Omnibus (GEO) database (accession number: GSE72050).

### Yeast one-hybrid assay

The NACRS motif (acacgcatgt) and the mutant motif (acacAcaCAC) were synthesized in four repeats. Both sequences were cloned into the bait vector pAbAi according to the procedure described in the MatchmakerTM Gold Yeast One-Hybrid Library Screening System user manual (Clontech, CA, USA). The complete CDS of *VaNAC26* was cloned into the prey vector pGADT7 AD. Then, the yeast strains that contained the bait and prey were cultivated on the SD/-Leu/-Ura/Aureobasidin A (AbA) media (200mg L^−1^ of AbA). The interaction between prey and bait was observed according to the growth of yeast strains.

### Quantification of JA

For WT and transgenic Arabidopsis, leaf tissues (200mg fresh weight) from WT, OE2 and OE3 plants were harvested under normal conditions. For grapevine, the plantlets were transferred to liquid 1/2 MS medium with 6% PEG 6000 to simulate water stress, and 200mg fresh weight of leaves were sampled at 0, 1, and 2 d after initiating water stress. JA was extracted and quantified by LC-MS/MS as described previously by Fu *et al.* (2012).

## Results

### *VaNAC26* contains a typical NAC domain in its N-terminal localized in the nucleus

The CDS of *NAC26* was cloned from *V. amurensis* and named *VaNAC26*. Compared with its homologous genes from ‘Pinot Noir’ (*GSVIVT01019952001*), only two single nucleotide polymorphisms (SNPs) were identified in the CDS of *VaNAC26* (Supplementary Fig. S1). The same deduced amino acid sequences were found in *VaNAC26* and *GSVIVT01019952001*.

**Fig. 1.**
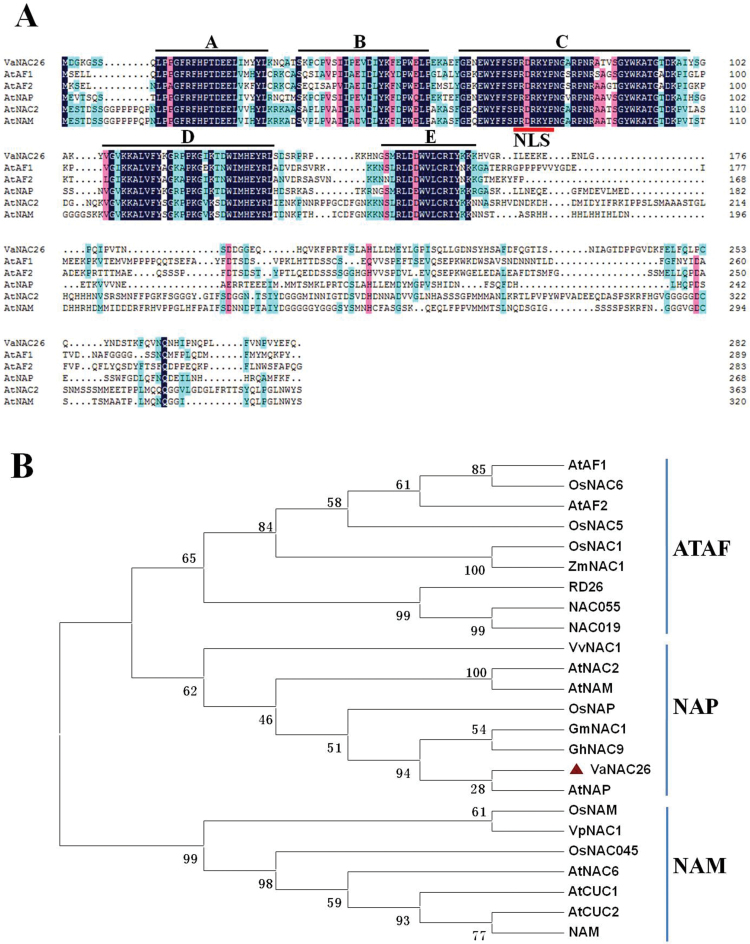
Sequence analysis of VaNAC26. (A) Multi-sequence alignment of VaNAC26 with several typical NAC proteins, including ATAF1 (GenBank accession no. NP_171677), ATAF2 (GenBank accession no. CAA52772), AtNAM (GenBank accession no. AAD17314), AtNAC2 (GenBank accession no. BT004079) and AtNAP (GenBank accession no. AJ222713) from Arabidopsis. Letters (A–E) above the sequences represent five conserved NAC subdomains. NLS represents nuclear localization signal. (B) Phylogenetic relationship between VaNAC26 and homologous proteins and other abiotic stress related NAC proteins. (This figure is available in colour at *JXB* online.)

The deduced protein sequence of *VaNAC2*6 contained 282 amino acid residues. Based on the multi-alignment of VaNAC26 with five NAC proteins from Arabidopsis, a typical highly conserved NAC domain (from 9 to 134 amino acid residues) was found in its N-terminal region and could be divided into five subdomains (A–E) according to Kikuchi *et al.* (2000) (Fig. 1A). The C-terminal region of VaNAC26 showed no significant similarity to any other members of the NAC family and represented a more variable region. The nuclear localization signal (NLS:PRDRKYP) was identified in the third motif of the NAC domain (Fig. 1A). A phylogenetic analysis was performed between VaNAC26 protein and other NAC domain-containing proteins that have been reported to be stress-related NACs. As shown in Fig. 1B, 24 NAC proteins could be clustered into three subgroups including ATAF, NAP, and NAM subgroups. VaNAC26 belongs to the NAP subgroup and showed highest similarity with AtNAP. VvNAC1, which regulates abiotic and biotic stress tolerances in grapevines, was also classified into this subgroup. NAC proteins that belong to NAP subgroups were found participating in responses to abiotic stresses in several species such as rice (Chen *et al.*, 2014; Liang *et al.*, 2014), grapevine (Le Hénanff *et al.*, 2013) and potato (Xu *et al.*, 2014).

**Fig. 2.**
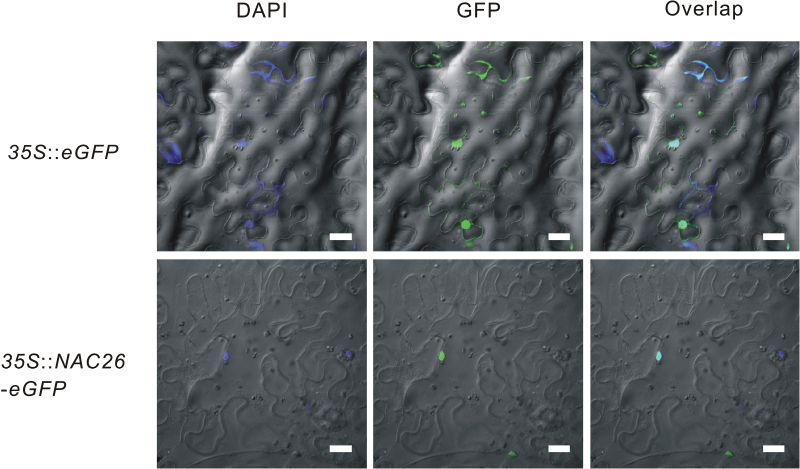
Subcelluar localization of *VaNAC26* in tobacco epidermis. *Nicotiana benthamiana* leaves were transiently infiltrated with *A. tumefaciens* GV3101 containing vectors expressing *35S::eGFP* and *35S::VaNAC26-eGFP*. Confocal images of peeled epidermis were captured 72h after inoculation. DAPI images are shown in the left panels; GFP fluorescence images in the middle panels; and overlap images in the right panels. Scale bars are 20 µm. (This figure is available in colour at *JXB* online.)

In order to identify the subcellular localization of VaNAC26, a full-length cDNA of *VaNAC26* was cloned into the pCAMBIA1302 vector under the control of the cauliflower mosaic virus (CaMV) 35S promoter and ligated into BglII/SpeI site of enhanced GFP (eGFP), resulting in an in-frame fusion protein of the VaNAC26::eGFP. The empty vector with only eGFP derived from the 35S promoter was used as a control. 4′, 6-diamidino-2-phenylindole (DAPI) was used during microscopic observation to show the nucleus region. As shown in Fig. 2 (upper panel), the tobacco epidermal cell only expressing GFPs showed cytoplasmic and nuclear staining, while VaNAC26::eGFP fusion protein displayed strong fluorescence in the cell nucleus region, which coincided with the DAPI stain result (Fig. 2, bottom panels). These results indicated that VaNAC26 is localized to the nucleus.

### VaNAC26 functions as a transcriptional activator with two activation regions

**Fig. 3.**
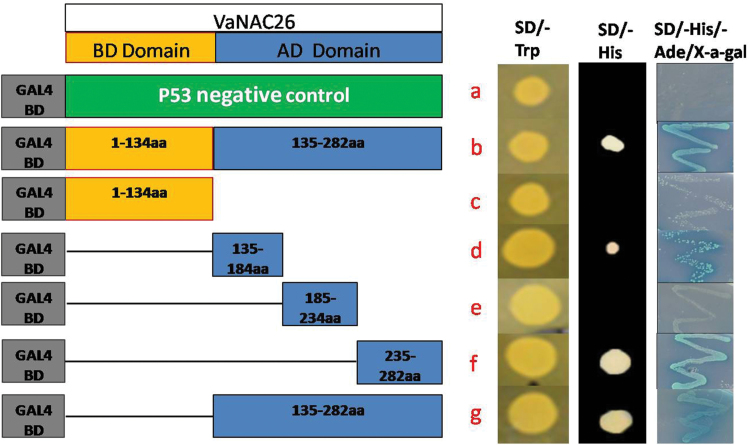
Transactivation assay of VaNAC26 in yeast. The fusion proteins of the GAL4 DNA-binding domain and VaNAC26 were expressed in yeast strain Y2HGold. Truncated VaNAC26 were fused with GAL4 BD (c–g), the vector pGBKT7-*P53* was used as negative control (a) and full-length VaNAC26 was fused with GAL4 BD domain (b). The culture solution of the transformed yeast was streaked on a SD/-Trp solid medium, SD/-His solid plate and SD/-His/-Ade/-X-α-gal medium, as indicated. (This figure is available in colour at *JXB* online.)

The function of TFs depends on transcriptional regulation of downstream genes. Typically, NAC proteins share a conserved N-terminal NAC domain (~150 aa) and a divergent C-terminal transcriptional regulatory region (Puranik *et al.*, 2012). To identify the transcriptional activity of VaNAC26, a transient expression assay was performed in yeast using a GAL4-responsive reporter system. A total of six effector plasmids were designed, containing translational fusions between the GAL4-binding domain-coding region and the full part, the putative binding domain, the putative activation domain or the truncated activation domain of VaNAC26 (Fig. 3, left). The empty pGBKT7 vector with the *P53* gene ligated after the GAL4-binding domain-coding region was used as a negative control. Then, the constructs were transformed to Yeast Y2H Gold cells and streaked on SD/-Trp, SD/-His and SD-His/-Ade/X-α-gal plates (Fig. 3, right). The pGBKT7 vector carries the TRP1 nutritional marker to select successfully transformed yeast colonies. Three integrated reporter genes (*ADE2*, *HIS3*, and *MEL1*) were in the Y2HGold yeast strain. Yeast colonies can grow on SD/-His/-Ade dropout medium when *ADE2* and *HIS3* are activated, and when they express *MEL1* they turn visibly blue in the presence of the chromagenic substrate X-α-gal. The full-length and putative activation region of VaNAC26 had activation ability and showed β-galactosidase activity (Fig. 3, b, g). The putative binding domain of VaNAC26, which contained the conserved NAC domain (A–D), did not promote yeast growth on SD/-His medium (Fig. 3, c). In the putative activation regions of VaNAC26, the activation ability was found in two independent regions (Fig. 3, d, f). One was located in the middle of VaNAC26 that contained the conserved NAC domain E (alkaline peptides, Supplementary Table S2), and the other was located near the C-terminal of VaNAC26 (acidic peptides, Supplementary Table S2). Both domains are stable hydrophilic peptides (Supplementary Table S2). These results indicated that VaNAC26 is an active transcriptional activator in yeast and two independent activation domains are located in the middle and C-terminal regions.

### *VaNAC26* showed quick and robust responses to low temperature, drought, and high salinity stresses and exogenous ABA treatment

**Fig. 4.**
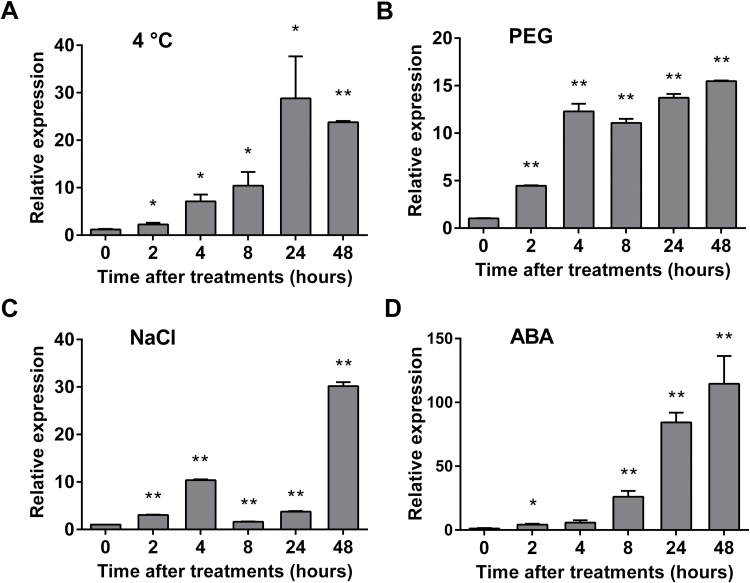
Expression patterns of *VaNAC26* under different stress and chemical treatments. *VaNAC26* relative expression under 4 ^o^C (A), 6% PEG (B), 100mM NaCl (C) and 100 μM ABA (D) treatments. The values represent the mean value ±SE from three replicates. * and ** indicate significant differences in comparison with values at 0h at *P*<0.05 and *P*<0.01 (*t*-test), respectively.

In our previous work, the public microarray data showed that the expression of *VvNAC26* was highly induced under abiotic stress conditions (Wang *et al.*, 2013). The responses of *VaNAC26* to low temperature, drought, and higher salinity stresses were investigated in this study. Plantlets of *V. amurensis* were exposed to stress conditions and qRT-PCR was performed. As shown in Fig. 4A, low temperature (4 ^o^C) induced *VaNAC26* transcripts in *V. amurensis*, and the highest expression level occurred 24h after the plants were subjected to cold treatment. Under an osmotic stress imitating drought treatment (PEG 6%), *VaNAC26* was upregulated shortly after the plantlets were subjected to water stress (2h), and the expression level increased over 10-fold at 4, 8, 24 and 48h after initiation of the treatment (Fig. 4B). The expression of *VaNAC26* significantly increased in plants only at 4h and 48h after subjecting them to high salinity stress (Fig. 4C). These results indicate that the expression level of *VaNAC26* can be induced quickly and intensively by abiotic stresses. ABA has been widely reported as an essential phytohormone in the regulation of abiotic stress-related signal pathways (Shinozaki and Yamaguchi-Shinozaki, 2007) As shown in Fig. 4D, the expression of *VaNAC26* increased continuously and up to 114.6-fold at 48h after exogenous ABA treatment, which indicated that the response of *VaNAC26* under abiotic stress conditions may be modulated by ABA-related signals.

### Heterologous overexpression of *VaNAC26* improved drought and high-salinity tolerances in Arabidopsis

To further investigate the function of *VaNAC26*, the CDS of this gene was transformed into Arabidopsis Col-0 WT plants under the control of the CaMV 35S promoter. The expressions of *VaNAC26* in homozygous T3 lines were confirmed by qRT-PCR (Supplementary Fig. S2). Three transgenic lines named OE-1, 2 and 3 were selected for the following analysis. The transgenic lines showed normal growth compared with WT plants (Supplementary Fig. S2), indicating that the overexpression of *VaNAC26* did not affect the main developmental processes in Arabidopsis.

**Fig. 5.**
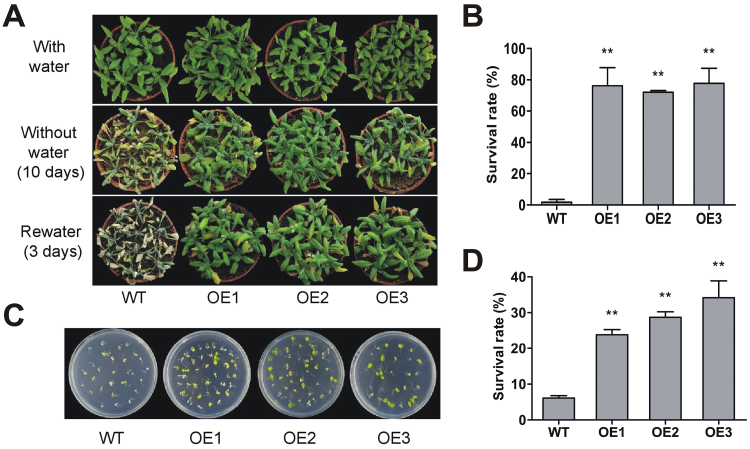
Drought and salt tolerance characterization of 35S::*VaNAC26* Arabidopsis. (A) Drought treatment and recovery of WT and three transgenic lines. (B) Survival rate of Arabidopsis under drought treatment. (C) Salt treatment: WT and three transgenic lines in plates with 1/2 MS medium supplemented with 120mM NaCl. (D) Survival rate of Arabidopsis under salt treatment. ** indicates significant differences in comparison with WT at *P*<0.01 (*t*-test). (This figure is available in colour at *JXB* online.)

The seedlings of WT and OE-1, 2 and 3 lines were subjected to low temperature, drought, and high-salinity treatments to investigate the functions of *VaNAC26* during abiotic stress responses. Although the expression of *VaNAC26* dramatically increased under low temperature in *V. amurensis*, no obvious differences were found between WT and transgenic lines when subjected to cold (data not shown). For the drought treatment, plants were grown in the greenhouse for 10 d without irrigation. As shown in Supplementary Fig. S3A, no significant differences were found between WT and the three transgenic lines in soil water content during the entire period of drought treatment, suggesting the intensity of drought stress in WT plants was similar to that of transgenic lines. The leaves of WT plants became yellow and wilted at 10 d (Fig. 5A), and most of them died after re-watering (survival rate: 1.75%; Fig. 5B). The transgenic lines showed greener leaves and a nearly 70% survival rate after re-watering (Fig. 5A, B). For high-salinity tolerance assessment, 7-day-old seedlings of WT and *VaNAC26*-OE lines were transferred to fresh 1/2 MS solid medium containing 120mM NaCl. The growth of most WT seedlings was inhibited, with yellow or white leaves after 7 d of salinity treatment (survival rate: 6% at 15 d of salinity treatment). By contrast, some of the OE-1, 2, and 3 plants continued growing under the high-salinity conditions (Fig. 5C, D). The survival rates of the OE-1, 2, and 3 lines were 24%, 29%, and 34%, respectively, which was significantly higher than that of WT plants (Fig. 5D). These data suggest that overexpression of *VaNAC26* increased the drought and high-salinity tolerances in Arabidopsis.

**Fig. 6.**
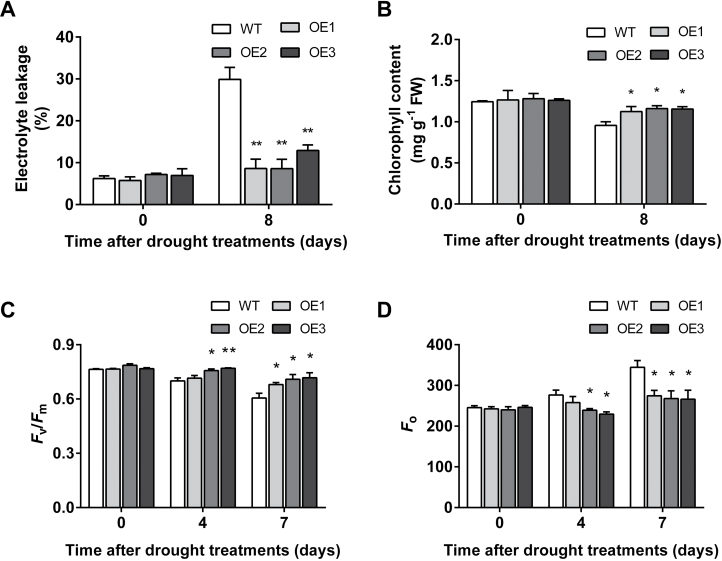
Analysis of electrolyte leakage (EL) (A), chlorophyll content (B), *F*_v_/*F*_m_ (C) and *F*_o_ (D) of WT and *VaNAC26*-OE lines. The EL (A) and chlorophyll (B) content were measured at 0 and 8 d after drought treatment, and *F*_v_/*F*_m_ (C) and *F*_o_ (D) were measured at 0, 4 and 7 d after drought treatment. The values in A/B and C/D represent the mean value ±SE from three and four replicates, respectively. * and ** indicate significant differences in comparison with WT at *P*<0.05 and *P*<0.01 (*t*-test), respectively.

In view of the remarkably improved drought tolerance in the OE lines, further studies were performed to investigate whether *VaNAC26* was involved in protecting the stabilities of cell membranes and/or the photosystem under drought stress conditions. Electrolyte leakage (EL), which is negatively correlated with cell membrane stability, was similar between WT and OE lines under well-irrigated conditions (Fig. 6A). However, after 8 d of drought treatment, the OE lines showed significantly lower EL than wild type plants (Fig. 6A). A significantly higher content of chlorophyll was also observed in OE lines than in WT plants after drought treatment (Fig. 6B). Chlorophyll fluorescence measurements reflect the susceptibility of photosystem II (PSII) to damage (Maxwell and Johnson, 2000). As shown in Fig. 6C, the maximum photochemical quantum yield of PSII (*F*_v_ */F*_m_) in transgenic lines was higher than in WT plants after 4 and 7 d of drought treatment (except for OE1 at 4 d). In contrast, a lower minimum fluorescence level (*F*_o_) was observed in transgenic lines at 4 and 7 d of drought treatment (except for OE1 at 4 d) (Fig. 6D). These results suggest that overexpression of *VaNAC26* increased the stabilities of cell membranes and PSII in transgenic Arabidopsis under water stress. Interestingly, there was no obvious difference in stomatal conductance (*g*_s_) between WT and OE under 4 and 7 d of drought treatment (Supplementary Fig. S3B), suggesting *VaNAC26* did not lower the transpiration rate so as to improve drought tolerance.

### ROS scavenging system was enhanced in *VaNAC26*-OE lines

**Fig. 7.**
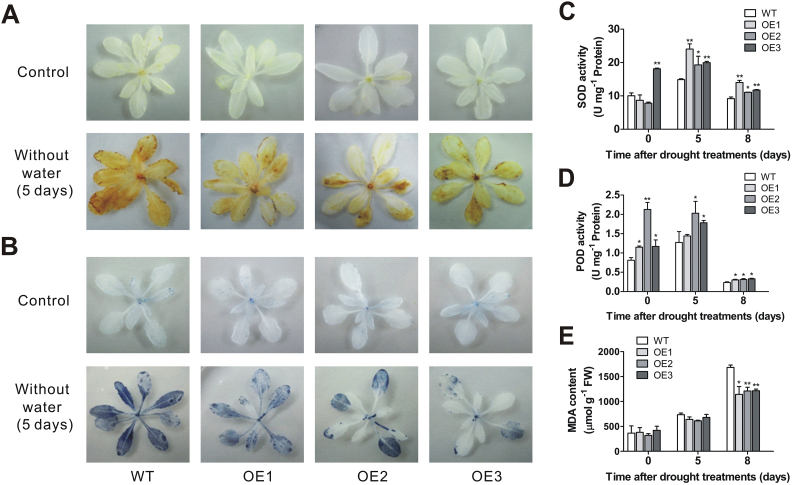
H_2_O_2_ and O_2_^−^ detection, antioxidant enzymes, and lipid peroxidation assay of WT and *VaNAC26*-OE lines. (A) H_2_O_2_ detection in WT and transgenic seedlings by DAB staining under normal conditions (upper) and 5 d after initiating drought treatment (lower). (B) O_2_^−^ detection in WT and transgenic seedlings by NBT staining under normal conditions (upper) and 5 d after initiating drought treatment (lower). The SOD (C) and POD (D) activities, and MDA content (E) of WT and three transgenic lines at 5 and 8 d after initiating drought treatment as well as normal conditions. The values represent the mean value ±SE from three replicates. * and ** indicate significant differences in comparison with WT at *P*<0.05 and *P*<0.01 (*t*-test), respectively. (This figure is available in colour at *JXB* online.)

To further illustrate the functions of *VaNAC26* in regulating tolerance to drought stress, physiological indices related to ROS scavenging such as the activities of the antioxidant enzymes SOD and POD, and H_2_O_2_, O_2_^−^_,_ and MDA content, were measured in the three OE lines under normal and drought conditions. DAB and NBT staining revealed that the presence of H_2_O_2_ and O_2_^−^ was rare in both wild type and transgenic seedlings under normal growth conditions (Fig. 7A, B). However, lower brown and blue staining intensities were observed in transgenic lines than in wild type plants at 5 d after initiating drought, suggesting lower H_2_O_2_ and O_2_^−^ content in the transgenic lines. Under normal conditions, the SOD activity in the OE3 line was significantly higher than in wild type plants (Fig. 7C). At 5 and 8 d after starting the drought treatment, all three transgenic lines showed higher SOD activities than wild type plants. The activities of POD in transgenic lines were higher than wild type under well-irrigated conditions, as well as at 5 or 8 d, except for OE1 line at 5 d, after starting the drought treatment (Fig. 7D). No obvious difference in MDA content was observed between wild type and transgenic lines within the first 5 d under drought stress (Fig. 7E). However, the three transgenic lines displayed significantly lower MDA content than the wild type at 8 d of drought stress. Overall, lower MDA, H_2_O_2_, and O_2_^−^ accumulation and higher activities of SOD and POD in transgenic lines indicated that *VaNAC26* increased drought tolerance in Arabidopsis by enhancing the ROS scavenging system.

### Genes related to the ROS scavenging system and JA biosynthesis were upregulated in *VaNAC26*-OE lines

**Fig. 8.**
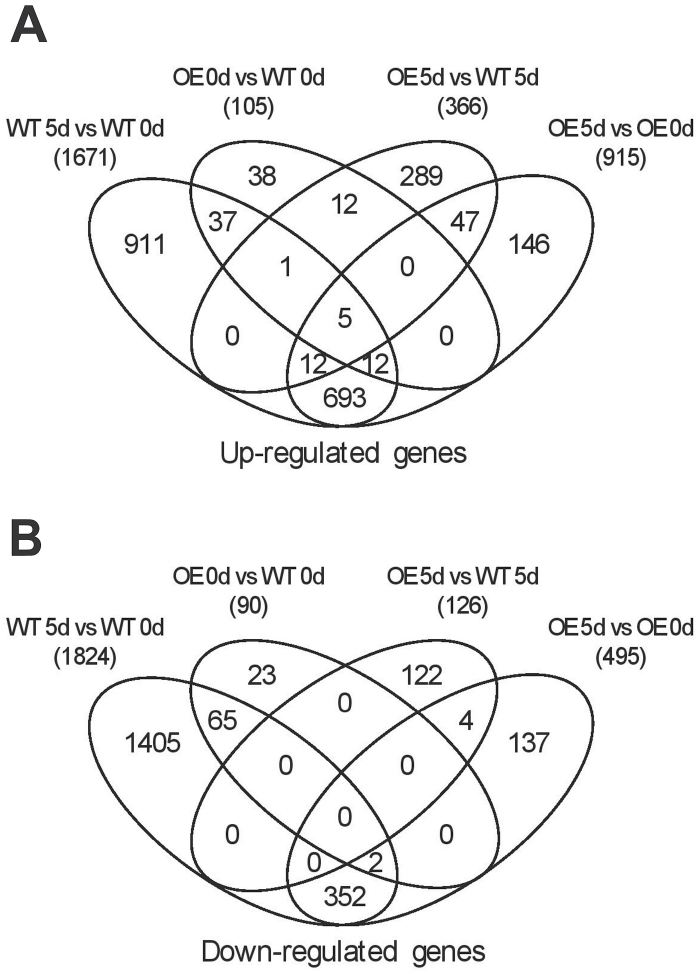
Venn diagram representations of the differentially expressed genes in four types of comparisons in WT and *VaNAC26*-OE plants under normal conditions and drought stress. (A) and (B) show the numbers of overlapping upregulated and downregulated genes, respectively. The numbers in brackets represent the total numbers of differentially expressed genes in different comparisons.

To investigate the possible roles of the *VaNAC26* at the transcription regulation level, a genome-wide expression profile analysis was performed using GeneChips. Samples of the three transgenic lines were pooled in equal proportions, and transcription profiles were compared with wild type Arabidopsis under normal conditions or at 5 d after initiating drought stress. The expressions of 195 genes were significantly changed in the *VaNAC26*-OE lines under normal growth conditions compared with wild type plants. Among them, 105 genes were upregulated while 90 genes were downregulated (OE 0d vs WT 0d, Fig. 8, Supplementary Table S3). These genes represented the candidate downstream genes regulated directly or indirectly by *VaNAC26*. On the 5th day under drought stress, a total of 1671 upregulated genes and 1824 downregulated genes were identified in wild type plants (WT 5d vs WT 0d, Fig. 8), while fewer changed genes were found in the *VaNAC26*-OE lines, with only 915 upregulated genes and 495 downregulated genes (OE 5d vs OE 0d, Fig. 8). When comparing gene expression data of OE lines with wild types under drought stress, a total of 492 differentially expressed genes were identified (OE 5d vs WT 5d, Fig. 8). Commonly overlapped genes showing similar changes in the four comparisons are presented in Fig. 8, and the details of the genes’ names and their functional annotation are listed in Supplementary Table S3. Five upregulated genes were identified in all four of the comparisons mentioned above (first five lines in Supplementary Table S3). Among them, one dehydrin (*LTI30*) and two *COR* genes (*COR15A* and *COR414-TM1*) are widely regarded as stress-related genes that respond to multiple abiotic stresses such as drought and cold (Baker *et al.*, 1994; Welin *et al.*, 1994; Breton *et al.*, 2003).

Pathway enrichment analysis revealed that the expression of many genes involved in diverse pathways were upregulated by the *VaNAC26* transgene and drought, including those involved with metal handling, stress, development and several other metabolic pathways involving nucleotides, amino acids, secondary products, hormones, and major carbohydrates (CHO) (Table 1, group I). Only two pathways, stress and hormone metabolism, were consistently greater by at least 2-fold normalized frequency values in all four comparisons (Table 1, group I). Interestingly, pathways including redox and transport were over-represented in OE plants compared with WT under normal conditions, but they were under-represented at the 5th day under drought treatment (Table 1, group II). In addition, the protein pathway was under-represented in all four comparisons (Table 1, group III).

**Table 1.** Pathway enrichment analysis of four types of comparisons from WT and OE microarrays under normal and drought stress conditions.

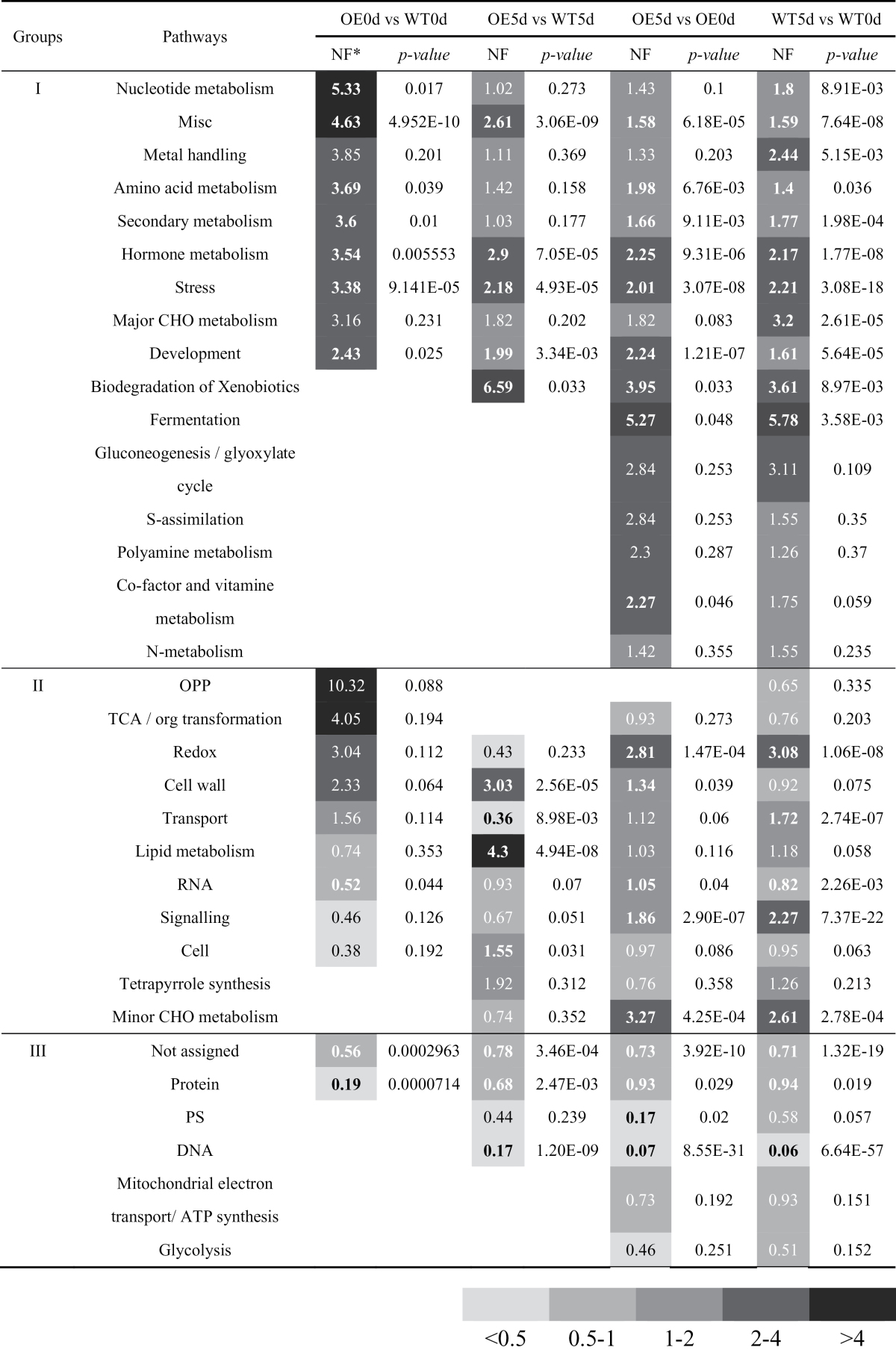

* NF, normalized frequency = sample frequency of given pathway category in this experiment/ background frequency of given pathway category in the ATH1 array.

The numbers in bold represented NF values with *P*-values <0.05.

To confirm the microarray results, qRT-PCR was conducted for 11 genes that showed differential expression in the OE lines and wild type plants in normal and drought conditions (Supplementary Fig. S4). All these genes showed similar expression changes between microarray and qRT-PCR data, which indicates the reliability of the microarray-based transcription profiles analysis.

Table 2 shows 20 differentially expressed genes in the *VaNAC26*-OE lines compared with wild type plants under normal conditions. The functional annotation by GO analysis indicated that these genes are all stress-related. Among these genes, the increased transcript levels of *SOD* (At4g25100) and *POD* (At3g45140 and At3g42570) in transgenic lines coincided with the results of ROS scavenging detection and histochemical staining (Fig. 7). Interestingly, JA biosynthetic related genes, such as *LOX2* (At3g45140), *AOS* (At5g42650), and *AOC1* (At3g25760) (Sasaki-Sekimoto *et al.*, 2013), were upregulated in the *VaNAC26*-OE line. Several marker genes in JA-related signal pathways including *PDF1.2* (At5g44420), *PDF1.2b* (At2g26020), *THI2.1* (At1g72260) (Xu *et al.*, 2001), *MYC2* (At1g32640), and *VSP1* (At5g24780) also showed significant changes. The expression of *PDF1.2*, for example, increased over 17-fold in transgenic lines relative to wild type plants. These results showed the enhancements of JA synthesis and the JA signal pathway in *VaNAC26*-OE lines.

**Table 2.** Transcripts highly upregulated in *VaNAC26* transgeneic Arabidopsis compared with WT under normal conditions.

AGI	Gene Annotation	Gene Symbol	Fold change	Gene Function	NACRS*
At5g44420	Antifungal like protein	PDF1.2	17.23	Defense and stress response	2
At4g15210	Beta-amylase	BAM5	11.31	Carbohydrate metabolic process	2
At2g26020	Putative antifungal protein	PDF1.2b	7.59	Defense and stress response	2
At4g23600	Tyrosine transaminase like protein	CORI3	5.76	Plant metabolic process	2
At5g24780	Vegetative storage protein	VSP1	5	Defense response to abiotic and biotic stress	2
At3g25760	Hypothetical protein	AOC1	3.82	Hormone biosynthetic process and stress response	3
At3g45140	Lipoxygenase	LOX2	3.25	Plant biosynthetic and metabolic process and stress response	4
At5g05340	Peroxidase	–	2.99	Response to oxidative stress	2
At1g18710	Myb-related transcription factor	AtMYB47	2.81	Defense and stress response	3
At1g19640	Floral nectary-specific protein	JMT	2.8	Hormone biosynthetic process and stress response	3
At2g42540	Cold-regulated protein cor15a precursor	COR15A	2.8	Defense response to abiotic and biotic stress	1
At1g52890	NAM-like protein	ANAC019	2.77	Defense response to abiotic and biotic stress	3
At1g29395	Expressed protein	COR414-TM1	2.61	Response to abiotic stress	3
At5g42650	Allene oxide synthase	AOS	2.61	Plant biosynthetic and metabolic process and stress response	0
At1g32640	Protein kinase, putative identical to bHLH protein	MYC2	2.45	Plant development, hormone biosynthetic process and stress response	1
At3g42570	Putative protein peroxidase	—	2.41	Response to oxidative stress	3
At1g72260	Thionin	THI2.1	2.4	Defense response	2
At4g25100	Superoxide dismutase (Fe)	FSD1	2.26	Response to oxidative stress	2
At4g37150	Hydroxynitrile lyase like protein	MES9	2.19	Plant metabolic process and stress response	1
At3g50970	Dehydrin Xero2	LTI30	2.01	Defense and stress response	1

* Numbers of putative NACRS motifs in the 1kb promoter of each gene.

### NACRS motif accumulated in upregulated genes in *VaNAC26*-OE lines and could be bound by VaNAC26 in yeast

**Fig. 9.**
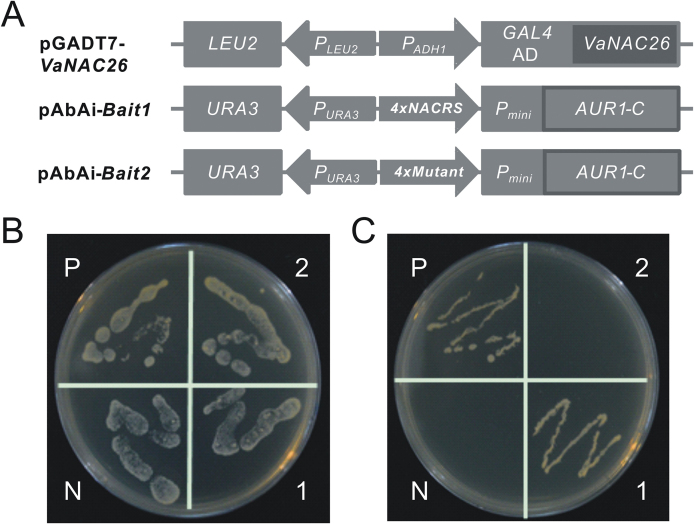
Yeast one hybrid assay of interaction of VaNAC26 on NACRS and its mutation sequence. Yeast transformants were examined by growth performance on SD/-Leu/-Ura with 500mg L^−1^ of AbA. The pAbAi-*P53*-transformed Y1HGold was set as negative control and the pGADT7-*P53* plus pAbAi-*P53* transformants were set as positive control. (A) Structural illustration of pGADT7-VaNAC26 and pAbAi-bait vectors. (B) and (C) represent the co-transformed yeast cells growth conditions on SD/-Leu/-Ura media and SD/-Leu/-Ura with 500mg L^−1^ of AbA, respectively. P, positive control; N, negative control; 1, transformants with pGADT7-VaNAC26 and pAbAi-4×NACRS; 2, transformants with pGADT7-VaNAC26 and pAbAi-4×mutants.

In Arabidopsis, ANAC019, ANAC055, and ANAC072 binds to NACRS in the promoter of ERD1 (Tran *et al.*, 2004), and this binding specificity has been confirmed by many other NAC proteins (Puranik *et al.*, 2012). Among 20 stress-related genes that were upregulated in our OE lines (Table 2), 19 genes contain the NACRS core motif in their upstream 1.0kb promoter region (Table 2), while some genes were assumed to be a direct target of *VaNAC26*. To verify the NACRS-binding ability of VaNAC26, the coding region of VaNAC26 was ligated to the yeast expression vector pGADT7 to produce a recombinant plasmid pGADT7-VaNAC26, and a four tandem repeated NACRS motif (CACGCATGTG) and its mutant sequence (CAttttTGTG), which was substituted for four bases (lower letters) compared with NACRS, were ligated to pAbAi (Fig. 9A). AbA is a cyclic depsipeptide antifungal agent with activity against yeast cells (Takesako *et al.*, 1991). The AbA resistant gene *URA-3* was integrated into Y1HGold yeast by the pAbAi vector, and it was used as a reporter gene to screen for putative binding activity of protein–DNA interactions. The result (Fig. 9B) showed successfully transformed Y1Hgold grew on SD/-LEU/-URA medium, and only the positive control and those cotransformed with VaNAC26 and NACRS could grow on AbA-containing medium (Fig. 9C), indicating that VaNAC26 could bind to NACRS but not its mutant sequence.

### Endogenous JA content increased in *VaNAC26*-OE lines and drought-treated *V. amurensis*

**Fig. 10.**
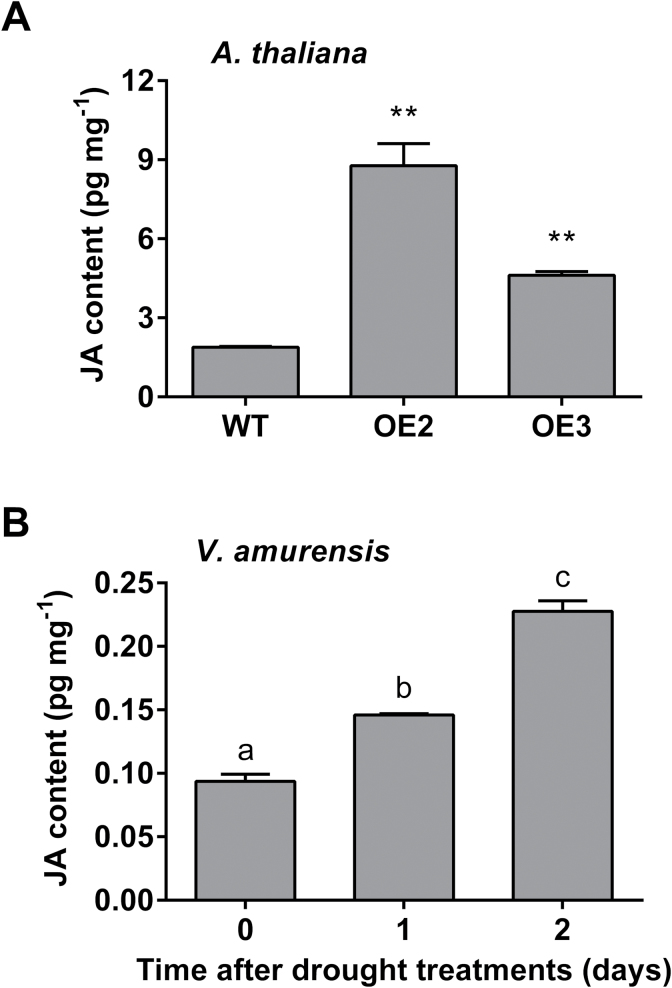
JA accumulation in Arabidopsis (WT and *VaNAC26*-OE lines) and drought-treated *V. amurensis*. (A) JA content in leaves of WT and two *VaNAC26*-OE lines (OE2 and OE3) under normal condition. (B) JA contents of *V. amurensis* expanded leaves after 0, 1 and 2 d of PEG 6% treatment. The values represent the mean value ±SE from three replicates. In (A) ** indicates significant differences in comparison with WT at *P*<0.01 (*t*-test). In (B) different letters indicate significant differences at *P*<0.05 (*F*-test).

JA is an important signaling molecule in a plant’s defense against biotic and abiotic stresses (Sasaki-Sekimoto *et al.*, 2005). Since there was increased transcription level of JA biosynthesis and signaling pathway-related genes in the *VaNAC26*-OE lines, we wondered whether the endogenous JA content changed in the transgenic lines. As shown in Fig. 10A, the wild type plants had 1.89±0.05 pg mg^−1^ fresh weight JA in their leaves while the JA content was up to 4.37±0.18 pg mg^−1^ in OE3 and 8.78±0.71 pg mg^−1^ in OE2. The endogenous JA content showed a significant increase in these two transgenic lines than in wild type plants under normal growth conditions (Fig. 10A). Combining the transcription profile data and the JA content analysis, we assumed that overexpression of *VaNAC26* in Arabidopsis enhanced JA synthesis, which may be responsible for the increased drought tolerance of the *VaNAC26*-OE lines.

To investigate the relationship between drought stress and endogenous JA level in *V. amurensis*, the JA content of *V. amurensis* leaf tissue was also measured under drought stress. A significant increase in JA level was observed in *V. amurensis* after the plants were subjected to drought treatment, and reached its highest level (2.4 fold increase) at 2 d (Fig. 10B). This result indicates that endogenous JA participates in the drought stress responses in *V. amurensis*.

## Discussion

*Vitis amurensis* is a wild species that survives the drought and cold winters in the Far East and North of China (Xin *et al.*, 2013). However, the underlying molecular mechanisms for these abiotic stress tolerance are not clear. In this study the possible roles of *VaNAC26* under abiotic stress were investigated using Arabidopsis transformation systems. Further work is still required to confirm the regulatory chain of *VaNAC26*, JA (synthesis and signaling), and tolerance (to drought) in grapevines.

Members of the *NAC* gene group that belong to the NAP subgroup have been reported to be closely associated with diverse biological processes including stress tolerance in plants. In Arabidopsis, an ABA-AtNAP-SAG113 (*SENESCENCE-ASSOCIATED GENE113*) regulatory chain was identified that controlled dehydration in senescing Arabidopsis leaves (Zhang and Gan, 2012). *OsNAP* was found to upregulate stress-related *OsPP2Cs* and TFs such as *OsDREB1A* and *OsMYB2* that improved drought and salt tolerances (Chen *et al.*, 2014). Heterologous expression of *VvNAC1* in Arabidopsis modified the transcription of defense marker genes such as *PDF1.2* and *VSP*, with enhanced abiotic and biotic stress tolerances (Le Hénanff *et al.*, 2013). The *VaNAC26* identified here also belongs to the NAP subgroup, and enhanced expression of *VaNAC26* in Arabidopsis might contribute to drought and salt tolerance. These results reinforce the essential roles of NAP subgroup genes in abiotic stress responses in plants.

Phytohormones such as SA, JA, ethylene, and ABA are known to control key signaling pathways in plant defense processes against biotic and abiotic stresses (Munné-Bosch and Müller, 2013). A previous study highlighted that the function of *NAC* genes may be divided into ABA-dependent and ABA-independent pathways based on the difference in their promoter elements (Purnaik *et al.*, 2012). In several species, exogenous SA, JA, and ethylene induced the expression of *NAC* genes (Bu *et al.*, 2008; Zhu *et al.*, 2012; Le Hénanff *et al.*, 2013). In addition, *NAC* genes were also found to provide feedback to the phytohormone signaling pathway by directly or indirectly regulating the synthesis of phytohormomes (Liang *et al.*, 2014). In this study, the expression of *VaNAC26* was induced by exogenous ABA application. However, based on microarray data, there were no significant differences in the expression of ABA biosynthesis genes between the *VaNAC26*-OE lines and WT (Supplementary Table S4). This indicates that *VaNAC26* does not improve drought tolerance via the ABA biosynthetic pathway, although *VaNAC26* was regulated by ABA. This result might explain the lack of a difference in ABA-induced stomatal conductance between WT and the *VaNAC26*-OE lines.

The connection between increased JA level and drought tolerance has been widely reported in many higher plants (Kazan, 2015). The content of endogenous JA generally increases in plants under water stress (Pedranzani *et al.*, 2007; Zhang and Huang, 2013), and exogenous application of JA (or methyl jasmonate, MeJA) at certain concentrations has been shown to improve drought tolerance (Mahmood *et al.*, 2012; Alam *et al.*, 2014). Here, we have demonstrated that these relationships also exist in *V. amurensis*, which also provides clues for subsequent analysis of ther *VaNAC26*-JA-drought tolerance regulatory pathway. JA signaling has been proved to be important for abiotic stress. For example, *OsbHLH148* constitutes the bHLH148-JAZ-COI1 pathway that affects JA signaling and improves drought tolerance in rice (Seo *et al.*, 2011), and several mutants involved in JA biosynthesis and signaling are sensitive to freezing stress in Arabidopsis under consistent or acclimation conditions (Hu *et al.*, 2013). In our study, several hormone-related genes were upregulated in the *VaNAC26*-OE lines, especially JA biosynthesis (*LOX2*, *AOS*, and *AOC1*, Table 2) and signaling-related genes (*PDF1.2*, *PDF1.2b*, *THI2.1*， *MYC2*, and *VSP1*, Table 2). NACRS motifs were detected in promoters of these genes (except *AOS*), indicating the possibility of direct targets of *VaNAC26*. An increased JA content was also found in *VaNAC26*-*OE* lines versus wild type Arabidopsis. These data suggest a putative role for *VaNAC26* in drought stress responses in grapevines by the *VaNAC26*-JA (synthesis and signaling)-tolerance (drought) regulatory network.

In model plants, *ANAC019* and *ANAC055* are thought to function as transcription activators to regulate JA-induced expression of defense genes (Bu *et al.*, 2008). *ANAC019* was also upregulated in our OE lines (Table 2). A homologue of *VaNAC26* in rice, *OsNAP*, was first reported to enhance JA biosynthesis and accumulation by improving the expression of *OsLOX2* and *OsAOC2* to regulate senescence (Zhou *et al.*, 2013). Moreover, *OsNAP* conferred drought and salt tolerances to rice through an ABA-dependent pathway (Chen *et al.*, 2014). Furthermore, *OsNAP* was specifically induced by ABA and regulated by ABA content via a feedback mechanism (Liang *et al.*, 2014). Here, we found that the expression of *VaNAC26* was induced by ABA but the increased drought-stress tolerance in *VaNAC26* overexpression lines may be related to JA-related signal pathways. Further work is still needed to determine how ABA triggers the accumulation of *VaNAC26* and how *VaNAC26* regulates its downstream genes to enhance the drought-stress responses in *V. amurensis*.

In grapevines, *VvNAC1* has been reported to respond to abiotic and biotic stresses as well as hormone treatment, and the transcriptional level of several marker genes (*PR-1*, *PDF1.2*, and *VSP1*) were enhanced in *VvNAC1*-overexpression lines in Arabidopsis (Le Hénanff *et al.*, 2013). These results indicate that more than one *NAC* member may be involved in the regulation of JA biosynthesis and downstream signaling pathways.

Besides involving the JA signaling response, overexpression in Arabidopsis resulted in less ROS accumulation and higher antioxidant enzymes activity, which was consistent with the microarray results that showed a higher expression of *SOD* and *POD* genes, indicating an important role for *VaNAC26* in ROS scavenging. Improvement of stress tolerance is often coupled with increased activities of antioxidant enzymes to remove harmful ROS (Xia *et al.*, 2015). Our results demonstrate that VaNAC26 plays an important role in drought, in part, by positively regulating SOD- and POD-mediated ROS scavenging. ROS scavenging is assumed to be associated with JA signaling and abiotic stresses in several species. For example, *TaAOC1* improved salt tolerance in wheat with increased JA accumulation and enhanced SOD activity (Zhao *et al.*, 2014). However, the detailed relationship between ROS scavenging and JA accumulation needs further study.

In our study, a remarkable response of *VaNAC26* to cold treatments in *V. amurensis* was also observed, which indicates an important role of *VaNAC26* in regulation of both cold and drought responses. However, there is no obvious phenotype in Arabidopsis OE lines under freezing treatments while drought tolerance is greatly enhanced, and a possible reason for this is that the transcription regulatory complex is composed of a series of members and some necessary factors are needed to activate genes involved in the cold response. In Arabidopsis, overexpression of *ANAC019*, *ANAC055*, and *ANAC072* activated *ERD1* by co-expressing *ZFHD1*, and *in vivo* analysis has shown protein–protein interactions between NAC proteins and ZFHD1 (Tran *et al.*, 2006), and ANAC096 and ABF2 synergistically activated the *RD29A* transcription (Xu *et al.*, 2013). The same study demonstrated that NAC proteins execute specific transcriptional regulation and may depend on co-operation with other factors to form a transcriptional complex. Hence, whether there are other factors synergistically functioning in cold tolerance in *V. amurensis* requires further study, and more clues may be found by genome expression profiles and co-expression analyses.

## Conclusions

In conclusion, the findings of this study indicate that *VaNAC26*, a member of the *NAC* genes that belong to the NAP subgroup, is responsive to abiotic stresses. It plays an important role in drought tolerance by regulating the expression of stress-associated genes, modulating JA synthesis and enhancing the antioxidant system. These findings demonstrate that *VaNAC26* constitutes an integral component of the drought-signaling network.

## Supplementary data

Supplementary data are available at *JXB* online.

Table S1. Sequence of qRT-PCR primer pairs used in this study.

Table S2. Features of two activation domains in VaNAC26 protein.

Table S3. Microarray-based differential expression gene analysis and its functional annotations in WT and *VaNAC26*-OE lines. (xls document).

Table S4. Microarray-based fold changes of ABA biosynthesis and ABA dependent gene expression between WT and *VaNAC26*-OE lines.

Figure S1. Comparisons of CDS regions between *VaNAC26* and *VvNAC26* (from ‘Pinot Noir’).

Figure S2. Growth conditions and expression detection of *VaNAC26* in WT and transgenic Arabidopsis.

Figure S3. Soil water content and stomatal conductance in WT and *VaNAC26*-OE lines under drought treatments.

Figure S4. Expression patterns of 11 putative *VaNAC26* downstream genes by qRT-PCR analysis.

Supplementary Data

## Abbreviations:

AREB/ABF: ABA-responsive element biding protein/ABRE-binding factor
CBF/DREB: C-repeat binding transcription factor/dehydration responsive element binding factor
eGFP: Enhanced green fluorescence protein
HD-ZIP: Homeodomain-leucine zipper
MDA: Malondialdehyde
MYB: v-myb avian myeloblastosis viral oncogene homolog
MYC: v-myc avian myelocytomatosis viral oncogene homolog
NAC: *N*AM (No apical meristem), *A*TAF 1/2 (Arabidopsis transcription activation factor 1/2), *C*UC 2 (Cup-shaped cotyledon 2)
NACRS: NAC recognition sequence, *cis*-element of NAC transcription factors.
