# Supplementary material for: Expression of Vitis amurensis NAC26 in Arabidopsis enhances drought tolerance by modulating jasmonic acid synthesis
Source: J Exp Bot. 2016 May 7;67(9):2829–45. doi: 10.1093/jxb/erw122 (PMC4861026; doi:10.1093/jxb/erw122)
Supplement: Supplementary Data [file supp_67_9_2829__index.html]

Expression of Vitis amurensis NAC26 in Arabidopsis enhances drought tolerance by modulating jasmonic acid synthesis — Supplementary Data 

# Expression of *Vitis amurensis NAC26* in Arabidopsis enhances drought tolerance by modulating jasmonic acid synthesis

## Supplementary Data

Data files

- supplementary\_tables\_S1\_S2\_\_S4\_\_figures\_S1\_S4.pdf - Supplementary Data
- supplementary\_table\_S3.xls - Supplementary Data
