# Supplementary material for: Expression of Vitis amurensis NAC26 in Arabidopsis enhances drought tolerance by modulating jasmonic acid synthesis
Source: J Exp Bot. 2016 May 7;67(9):2829–45. doi: 10.1093/jxb/erw122 (PMC4861026; doi:10.1093/jxb/erw122)
Supplement: Supplementary Data [file supp_erw122_supplementary_tables_S1_S2__S4__figures_S1_S4.pdf]

Title:

Expression of *Vitis amurensis NAC26* in Arabidopsis enhances drought tolerance by modulating jasmonic acid synthesis

Linchuan Fang[§], Lingye Su[§], Xiaoming Sun, Xinbo Li, Mengxiang Sun, Sospeter Karanja Karungo, Shuang Fang, Jinfang Chu, Shaohua Li[*], Haiping Xin[*]

Supplementary data

Supplementary Table S1. Sequence of qRT-PCR primer pairs used in this study.

Name	Orientation	Sequence (5'-3')
VaNAC26	Forward	TGATTGGGTGCTATGTAGG
	Reverse	TTTCACTTGGTGCTGCTC
VvActin7	Forward	CTTGCATCCCTCAGCACCTT
	Reverse	TCCTGTGGACAATGGATGGA
VvGADPH	Forward	GGTATTAGGAACCCAGAGGAGA
	Reverse	CAACAACGAACATAGGAGCAT
PR5	Forward	GTGCTTGCTCTGCCTTCA
	Reverse	CTGGTTGGGTCGTCGTAA
MYB47	Forward	CGTTTGTGATTGGCGTTCC
	Reverse	GGCTGCCCACCTGTTTCC
COR414-TM1	Forward	GCTTAGGTTATCGGGTGA
	Reverse	CGGAGCAGCATAACAGAC
NATA1	Forward	GCACCAGAGCCAAACACT
	Reverse	TGGAGGCAAGACCAGACT
COR15A	Forward	CTTTCCACAGCGGAGCCA
	Reverse	TTGCCGTCACCTTTAGCG
SWEET4	Forward	GTTGCGAGAAACATTGCC
	Reverse	GCTTTGTACTCCTCCACCTT
14A	Forward	CCCCAACACGCAGCATAC
	Reverse	CGGCAATGTCCAAAGTCT
BMY1	Forward	CGTTGGGAGTTGTGAATG
	Reverse	GAACAGCGTTTTGTAGGC
PDF1.2	Forward	GCTTCCATCATCACCCTT
	Reverse	ATGTCCCACTTGGCTTCT
LTP4	Forward	TGGCACAGTGGCAAGTAG

	Reverse	GAGATGGGATAGGGGATG
LTP3	Forward	GGCTTTCGCTTTGAGGTT
	Reverse	TTGTTGGCGGTCTGGTGT
Actin2	Forward	TTACCCGATGGGCAAGTCA
	Reverse	AAACGAGGGCTGGAACAAGA
UBQ10	Forward	CACACTCCACTTGGTCTTGCGT
	Reverse	TGGTCTTTCCGGTGAGAGTCTTCA

Supplementary Table S2. Features of two activation domains in VaNAC26 protein

number	location	MV	pI	Stable index	Aliphatic index	GRAVY
Domain 1	135-184aa	5856.7	9.65	39.84	72.00	-1.176
Domain 2	231-282aa	5954.6	4.43	26.36	69.23	-0.523

135-184aa: SDSRPRPKKHNGSMRLDDWVLCRIYKKKHVGRILEEKEENLGPQIPVTNS

231-282aa: TISNIAGTDPPGVDKFELFQLPCQYNDSTKFQVNQNHIPNQPLFVNPVYEFQ

MV: molecular weight; pI: isoelectric point; GRAVY: grand average of hydropathicity.

Supplementary Table S3. Microarray-based differential expression gene analysis and its functional annotations in WT and *VaNAC26*-OE lines. (.xls document)

The numbers in red and green represent the fold changes of upregulated and downregulated genes in specific comparisons, respectively. The word ‘ns’ represents ‘no significant difference’.

Supplementary Table S4. Microarray-based fold changes of ABA biosynthesis and ABA dependent gene expression between WT and *VaNAC26*-OE lines.

AGI	Gene Symbol	OE 0d vs WT 0d	OE 5d vs WT 5d	AGI	Gene Symbol	OE 0d vs WT 0d	OE 5d vs WT 5d
ABA biosynthesis genes				At4g21440	ATMYB102	1.6021	1.2396
At4g18350	NCED2	1.2231	1.1054	At3g06490	MYB108	0.9918	0.9398
At3g14440	NCED3	1.1635	0.981	At5g67300	MYBR1	0.9365	0.8387
At1g30100	NCED5	1.1061	0.8603	At4g28110	AtMYB41	0.8697	1.1997
At3g24220	NCED6	1.1274	1.1122	At1g52890	ANAC019	**2.772**	1.0669
At1g78390	NCED9	0.9179	0.9968	At3g15500	ANAC055	0.831	1.2371
At5g67030	ABA1	1.3294	0.7967	At1g01720	ATAF1	0.9608	0.9888
At1g52340	ABA2	1.1299	1.1038	At3g15510	ATNAC2	0.7931	1.3876
At2g27150	AAO3	0.9799	0.7028	At1g69600	ZFHD1	0.9827	1.6189
ABA-dependent genes				At5g65310	ATHB5	0.8545	1.336
At1g49720	ABF1	1.3823	1.3075	At2g22430	ATHB6	1.3481	1.002
At4g34000	ABF3	1.0401	0.815	At2g46680	ATHB-7	1.4103	**2.0838**
At3g19290	ABF4	0.8286	0.9157	At3g61890	ATHB-12	1.219	1.591
At5g51990	CBF4	0.9459	0.9606	At1g32640	MYC2	**2.4517**	1.0255
At5g05410	DREB2A	0.8333	0.8343	At2g46510	ATAIB	1.1979	1.012
At1g43160	RAP2.6	**2.7856**	0.2972	At3g19580	AZF2	0.8282	1.1096
At3g20310	ERF7	1.0464	1.1586	At4g17870	SAZ	0.9039	1.289
At5g19330	ADAP	0.9306	1.0126	At1g54160	NF-YA5	1.0805	1.0221
At2g47190	MYB2	1.3774	0.7904	At2g33380	RD20	**3.0546**	1.3497
At5g62470	MYB96	1.13	1.3141	At4g27410	RD26	1.0585	1.1467

The numbers in red represent the fold change of upregulated genes (fold change >2).

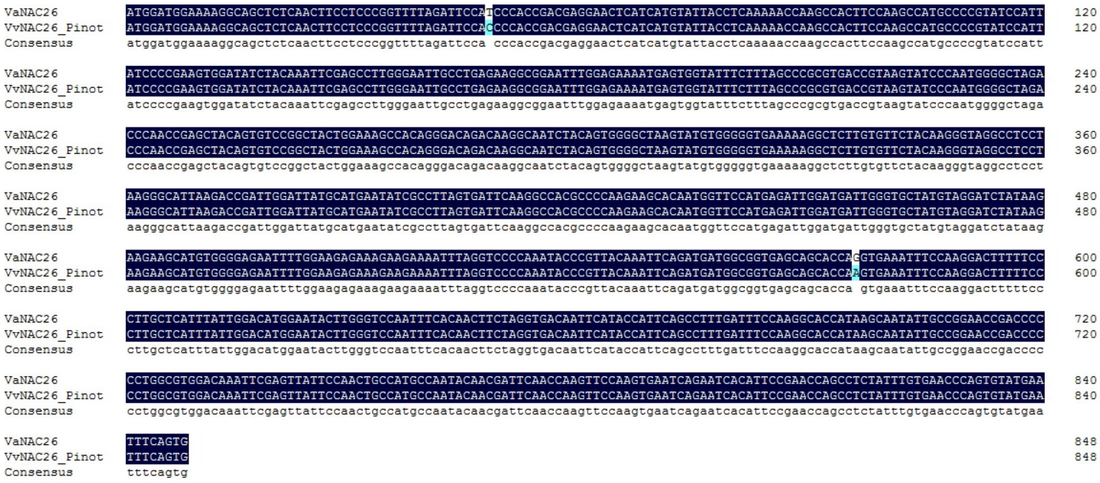

Supplementary Figure S1. Comparisons of CDS regions between *VaNAC26* and *VvNAC26* (from 'Pinot Noir').

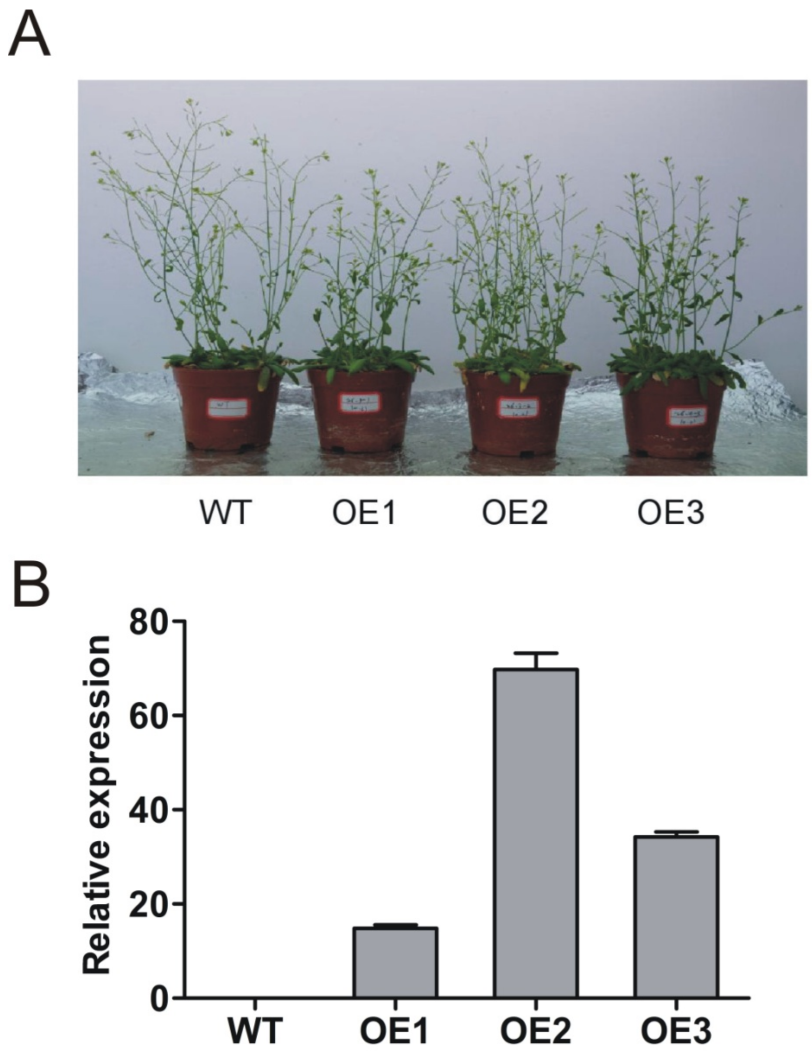

Supplementary Figure S2. Growth conditions and expression detection of *VaNAC26* in WT and transgenic Arabidopsis.

A: Growth of transgenic Arabidopsis shows identical phenotype at the same developmental stage. B: qRT-PCR detections of *VaNAC26* in WT and three *VaNAC26*-transgenic lines. The values represent the mean value ± SE from three replicates.

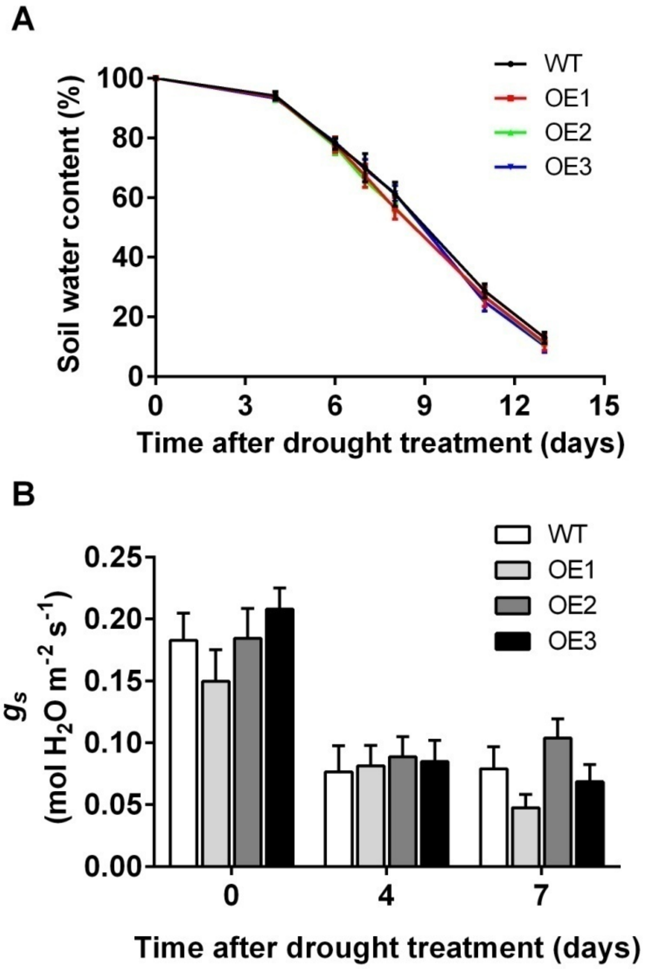

Supplementary Figure S3. Soil water content and stomatal conductance in WT and *VaNAC26*-OE lines under drought treatments.

Soil water content (A) was detected at different designated time intervals during drought treatments: 4 d (mild water stress), 6, 7, 8 d (moderate water stress), 11 and 13 d (severe water stress). Stomatal conductance (g_s, B) of expanded leaves was measured at 0, 4, and 7 d of drought treatment. The values in A and B represent the mean value ± SE from four and six replicates, respectively.

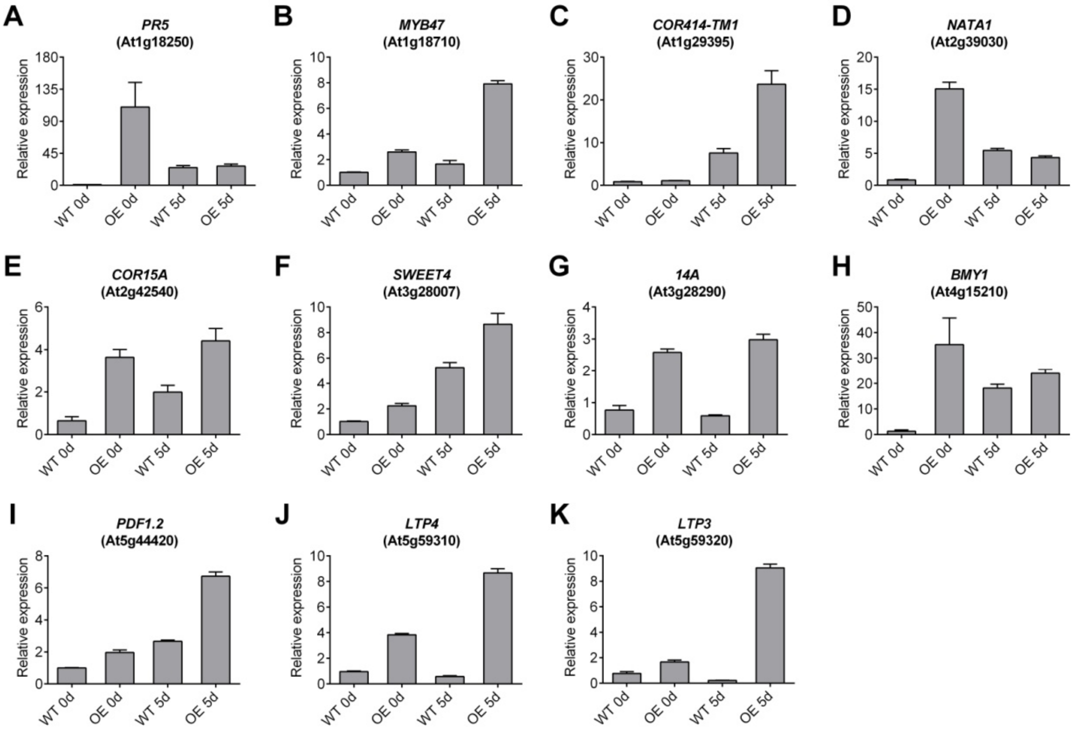

Supplementary Figure S4. Expression patterns of 11 putative *VaNAC26* downstream genes by qRT-PCR analysis.

Relative expressions of *PR5* (A), *MYB47* (B), *COR414-TM1* (C)， *NATA1* (D), *COR15A* (E) , *SWEET4* (F), *14A* (G) , *BMY1* (H) , *PDF1.2* (I) , *LTP4* (J) and *LTP3* (K) in WT and OE lines before (WT 0d and OE 0d) and 5 d after water stress (WT 5d and OE 5d), respectively. The values represent the mean value ±SE from three replicates.
